# Endonuclease EEPD1 Is a Gatekeeper for Repair of Stressed Replication Forks*

**DOI:** 10.1074/jbc.M116.758235

**Published:** 2017-01-03

**Authors:** Hyun-Suk Kim, Jac A. Nickoloff, Yuehan Wu, Elizabeth A. Williamson, Gurjit Singh Sidhu, Brian L. Reinert, Aruna S. Jaiswal, Gayathri Srinivasan, Bhavita Patel, Kimi Kong, Sandeep Burma, Suk-Hee Lee, Robert A. Hromas

**Affiliations:** From the ‡ Department of Biochemistry and Molecular Biology, Indiana University School of Medicine, Indianapolis, Indiana 46202,; the § Department of Environmental and Radiological Health Sciences, Colorado State University, Fort Collins, Colorado 80523,; the ¶ Department of Medicine and the Cancer Center, University of Florida Health, Gainesville, Florida 32610, and; the ‖ Department of Radiation Oncology, University of Texas Southwestern Medical Center, Dallas, Texas 75390

**Keywords:** DNA damage, DNA endonuclease, DNA repair, DNA replication, homologous recombination, replication fork stress, end resection, nuclease

## Abstract

Replication is not as continuous as once thought, with DNA damage frequently stalling replication forks. Aberrant repair of stressed replication forks can result in cell death or genome instability and resulting transformation to malignancy. Stressed replication forks are most commonly repaired via homologous recombination (HR), which begins with 5′ end resection, mediated by exonuclease complexes, one of which contains Exo1. However, Exo1 requires free 5′-DNA ends upon which to act, and these are not commonly present in non-reversed stalled replication forks. To generate a free 5′ end, stalled replication forks must therefore be cleaved. Although several candidate endonucleases have been implicated in cleavage of stalled replication forks to permit end resection, the identity of such an endonuclease remains elusive. Here we show that the 5′-endonuclease EEPD1 cleaves replication forks at the junction between the lagging parental strand and the unreplicated DNA parental double strands. This cleavage creates the structure that Exo1 requires for 5′ end resection and HR initiation. We observed that EEPD1 and Exo1 interact constitutively, and Exo1 repairs stalled replication forks poorly without EEPD1. Thus, EEPD1 performs a gatekeeper function for replication fork repair by mediating the fork cleavage that permits initiation of HR-mediated repair and restart of stressed forks.

## Introduction

The replicating cell perpetually suffers from DNA damage from both endogenous and exogenous sources. This DNA damage creates barriers for the replication fork, and replication is not a smooth, continuous process, but rather one of intermittent stress, with forks stalling and restarting (1–3). Timely, accurate fork restart is important for maintaining genome stability, and replication fork stress is a common precursor to genomic instability. This genomic instability often manifests as chromosome gain or loss, translocations, and mitotic catastrophe, with the cell dying, or worse, gaining tolerance of the chromosomal inequities, and transforming to a malignant phenotype (4–6). Thus, replication fork repair is a critical process, required to maintain genetic integrity.

Eukaryotic replication fork repair and restart are complex and incompletely understood. The homologous recombination (HR)^3^ repair pathway is thought to be responsible for high fidelity repair and restart of stressed replication forks (4–7). HR is initiated by 5′ end resection of free DNA double-strand (DS) ends to create 3′ single-stranded (SS) DNA, which then uses BRCA2/RAD51 and other HR factors to create heteroduplexes with homologous sequences, typically on sister chromatids (1, 2, 6–10). After the invading 3′ SS DNA is extended by DNA synthesis across the replication fork junction, these branched DNA structures may be resolved by either Gen1 or Mus81, with Slx4 serving as a scaffold (9–13).

End resection is not only the initial step in HR; it also represents a commitment to that repair pathway, and it directs the cell away from other repair pathways that generate genomic instability, such as classical or alternative non-homologous end-joining (NHEJ) (14–16). Both of these NHEJ pathways are non-conservative, resulting in at best deletions or insertions at the repair site, or at worst chromosomal fusions (17–22). When HR is defective, repair of stressed replication forks by unopposed NHEJ leads to chromosomal abnormalities (17, 18). Thus, 5′ end resection of stressed replication forks to initiate HR-mediated fork restart is essential to maintain the genome in its native state and to prevent transformation to malignancy.

End resection appears to have two phases, an initial short resection, followed by more extensive end resection (23, 24). Mre11 and BRCA1/CtIP play roles in initiating short end resection, while two protein complexes regulate long range end resection, one that includes the Dna2 nuclease, and a second centered on Exo1. Should long range end resection fail, the replication fork can still be rescued by alternative NHEJ, although this is non-conservative and can lead to chromosomal translocations (23, 25, 26). Unlike a DNA double-strand break, a stalled replication fork lacks a free DNA end from which one of the exonuclease complexes can initiate end resection (1–3, 14–16). A free double-strand end at a stressed replication fork can be created in at least two ways: the fork can regress into a “chicken foot structure” with a single DS end (27), or an endonuclease can cleave the fork, directly creating a free DS end upon which a 5′-exonuclease can act (11, 27, 28). Although there are candidates for such a stressed replication fork endonuclease, such as Mus81 and Gen1 (9, 29), there is controversy surrounding their function (10, 30). Mus81 is important in replication fork restart in mice, but was found to be less important in humans (10). The role of Gen1 in cleaving complex chromosomal structures may be restricted to mitosis, and therefore have little or no role in stressed replication fork repair (30). Thus, the identity of the endonuclease that can cleave replication fork structures to initiate end resection and fork repair remains undefined.

We recently reported that EEPD1 was important for HR and rescue of stressed replication forks (31), but its precise mechanism of action was not defined. As EEPD1 has N-terminal RvuA-like domains, an uncharacterized nuclease domain in the DNase I superfamily, and interacts with Exo1 (31), we hypothesized that EEPD1 could cleave replication fork structures to provide the free 5′ end to promote Exo1-mediated 5′ end resection (11, 28, 31). We report here that EEPD1 can indeed cleave replication fork structures to permit Exo1-mediated 5′ end resection and that both EEPD1 and Exo1 are required for proper replication fork restart after stalling.

## Results

### 

#### 

##### EEPD1 Is a 5′-Endonuclease That Can Cleave Replication Fork Structures

**FIGURE 1.**
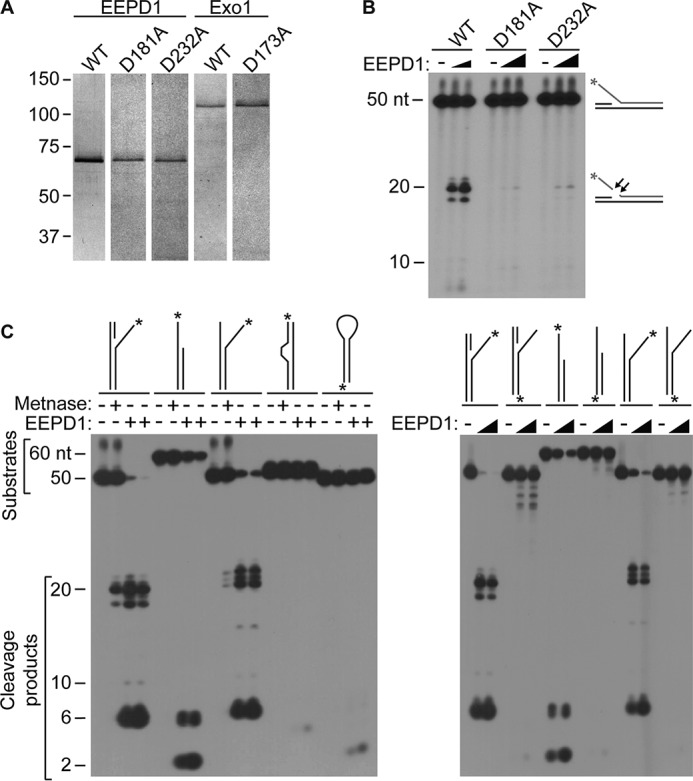
**EEPD1 is a structure-specific 5′-endonuclease.** *A*, representative silver-stained gel of purified EEPD1 and Exo1 protein. 0.1 μg each of EEPD1 and Exo1 were loaded onto the silver-stained gel. *B*, reaction products of WT EEPD1 or mutant (D181A and D232A) EEPD1 on 5′ SS flap substrate. 2 and 4 pmol of WT EEPD1 or mutant (D181A and D232A) EEPD1 were loaded on 240 fmol of 5′ ^32^P-labeled SS flap substrate. *C*, EEPD1 is a 5′ structure-specific endonuclease. Shown are nucleolytic cleavage products of various DNA structures by purified EEPD1 or Metnase, another 5′ structure-specific nuclease used as a positive control (32). Where indicated, 2 pmol of Metnase and 2 and 4 pmol of EEPD1 were used. 240 fmol of DNA substrates were used. Oligonucleotide substrates (32) in the *left panel* are 5′ SS flap (*lanes 1–4*), 5′ SS tail (*lanes 5–8*), 5′ SS pseudo-Y (*lanes 9–12*), bubble (*lanes 13–16*), and stem-loop (*lanes 17–20*). In the *right panel*, the substrates used are 5′ SS flap (*lanes 1–3*), 3′ SS flap (*lanes 4–6*), 5′ SS tail (*lanes 7–9*), 3′ SS tail (*lanes 10–12*), 5′ SS pseudo-Y (*lanes 13–15*), and 3′ SS pseudo-Y (*lanes 16–18*). * indicates 5′ ^32^P label.

We used purified recombinant EEPD1 (Fig. 1*A*) to characterize its nuclease activity on a variety of branched DNA structures *in vitro* (11, 12, 32, 33). EEPD1 cleaves 5′ SS flaps, and EEPD1 mutations (D181A or D232A) nearly abolished this activity (Fig. 1*B*), indicating that the observed nuclease activity with WT EEPD1 is not due to a contaminant (32, 33). We also found that EEPD1 cleaves 5′ SS overhangs near the SS-DS DNA junction, an activity similar to that of Metnase (Fig. 1*C*), another 5′-nuclease involved in replication fork restart (32–34). EEPD1 also cleaves 5′ SS flap and pseudo-Y structures near the SS-DS DNA junction, but it did not cleave bubble or stem-loop structures (Fig. 1*C*), suggesting that it is unlikely to be involved in VDJ recombination (11, 28, 35).

**FIGURE 2.**
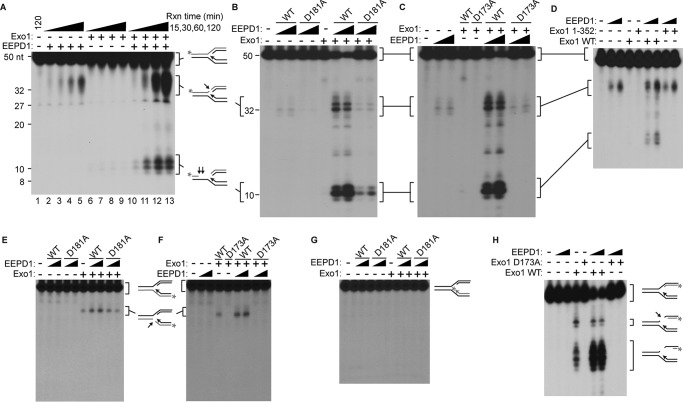
**EEPD1 cleavage of the replication fork parental lagging strand permits Exo1 5′ end resection.** *A*, time course of EEPD1 and/or Exo1 nucleolysis of lagging parental strand of a DS replication fork structure. * indicates 3′ ^32^P label. Where indicated, 2 fmol of Exo1 and 2 pmol of EEPD1 were used. 240 fmol of DNA substrates were used. *Rxn time*, reaction time. *B*, as in *panel A* with WT or nuclease-defective (D181A) EEPD1. *C*, as in *panel A* with WT or nuclease-defective (D173A) Exo1. *D*, truncated Exo1 with intact nuclease activity but without interaction with EEPD1 loses most exonuclease activity on the parental lagging strand. *E*, WT and nuclease-defective mutant EEPD1 (D181A) has no effect on the leading strand, whereas WT Exo1 has marginal endonuclease activity. *F*, nuclease-defective mutant Exo1 (D173A) loses the marginal endonuclease activity on the leading strand when compared with WT Exo1. *G*, WT or nuclease-defective mutant EEPD1 (D181A) with WT Exo1 has no activity on the daughter leading strand. *H*, WT but not nuclease-defective mutant Exo1 (D173A) with WT EEPD1 resects the daughter lagging strand. *Arrows* represent the EEPD1 site of action. Where indicated, 2 fmol of either WT or the mutant Exo1, and 2 and 4 pmol of either WT or the mutant EEPD1, were used. 240 fmol of DNA substrate were used.

Because EEPD1 is a 5′-endonuclease that is recruited to stressed replication forks and interacts with Exo1 (31), we hypothesized that EEPD1 could cleave replication fork structures to generate the needed free 5′-DNA ends upon which Exo1 can act (11, 12, 16, 24, 27, 28). We found that EEPD1 alone cleaves the lagging parental strand of a non-reversed replication fork structure at the bifurcation between parental and replicated strands (Fig. 2*A*). We then examined Exo1 exonuclease activity on parent and daughter leading and lagging strands of intact replication fork structures with and without EEPD1. We found that unless EEPD1 was present, Exo1 could not initiate 5′ end resection of the parental lagging strand (Fig. 2*A*). Indeed, Exo1 alone displayed little activity on the intact DS replication fork structure.

We then tested the effect of EEPD1 and Exo1 on 5′ end resection of the leading daughter strands. We found that, as with the parental strand, EEPD1 promoted Exo1's exonucleolytic resection of the lagging daughter strand. In contrast, there was no effect of either nuclease, alone or in combination, on the leading daughter strand.

When the D181A mutant of EEPD1, which has lost most of its endonuclease activity, replaced WT EEPD1, we found that the 5′-exonuclease activity of Exo1 on the parental lagging strand structure was markedly decreased (Fig. 2*B*). When the D173A mutant of Exo1, which has lost most of its nuclease activity, replaced WT Exo1, we discovered that the 5′ end resection of the parental lagging strand structure was also significantly decreased (Fig. 2*C*). Thus, the nuclease activities of both EEPD1 and Exo1 were necessary for proper 5′ end resection of the lagging strand of a replication fork structure. This also implies that the parental lagging DNA strand must be cleaved before Exo1 can initiate end resection at a replication fork structure, consistent with the Exo1 requirement for a free 5′ SS end (28).

##### Exo1 Interacts with EEPD1 and Improves Its Endonuclease Activity

When a truncation mutant of Exo1 that did not interact with EEPD1 was added to EEPD1, there was decreased 5′-exonucleolysis of the lagging parental strand structure (Fig. 2*D*). This implies that EEPD1 may assist in recruiting Exo1 to this replication fork structure and/or enhancing Exo1 nuclease activity. In addition, EEPD1 has little activity in cleavage of the leading parental strand of a replication fork structure (Fig. 2, *E* and *F*). Exo1 has a small amount of endonuclease activity on the leading parental strand (Fig. 2, *E* and *F*), likely due to its known weak 5′ flap nuclease activity (41). Nuclease-defective mutant Exo1 loses the marginal endonuclease activity on the leading strand when compared with WT Exo1. WT or nuclease-defective mutant EEPD1 with WT Exo1 has no cleavage or exonuclease activity on the daughter leading strand (Fig. 2*G*). WT but not nuclease-defective mutant Exo1, when present with WT EEPD1, resects the daughter lagging strand (Fig. 2*H*). Significantly, this implies that the parental lagging strand must be cleaved by EEPD1 prior to Exo1 5′ end resection of the daughter lagging strand.

**FIGURE 3.**
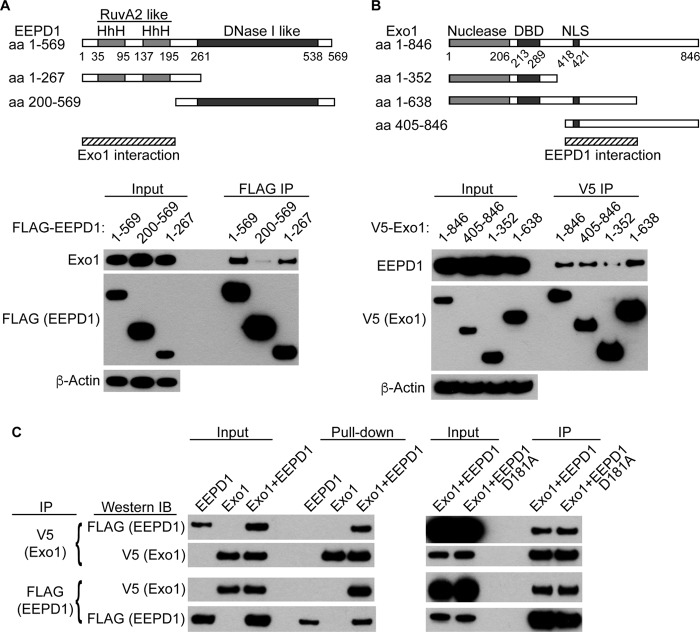
**Co-immunoprecipitation of EEPD1 and Exo1.** *A*, full-length and truncated versions of FLAG-tagged EEPD1 were expressed in HEK-293 cells and then immunoprecipitated with FLAG antibody, and Western blots were probed with antibodies to Exo1, FLAG, and β-actin. *aa*, amino acids. *HhH*, helix-hairpin-helix domain. *B*, full-length and truncated versions of V5-tagged Exo1 were expressed in HEK-293 cells and immunoprecipitated with V5 antibody and then Western blots were probed with antibodies to EEPD1, V5, and β-actin. Minimal interaction regions are shown below maps in *panels A* and *B* (*hatched bars*). *DBD*, DNA binding domain; *NLS*, nuclear localization signal. Initial immunoprecipitation was from 300 μg of total cell lysate, and the input was 3% of this. *C*, *in vitro* co-immunoprecipitation of FLAG-tagged EEPD1 with V5-tagged Exo1. The D181A mutant of EEPD1 still interacts with Exo1. Initial immunoprecipitation was from 200 ng of each protein, and the input was 2% of this. *IB*, immunoblot. Initial immunoprecipitation for the EEPD1/D181A and Exo1 interaction was as for *B*.

We then defined general interacting regions of Exo1 and EEPD1 using co-immunoprecipitation of transfected full-length and truncated protein species in cells. We found that deletion of the N-terminal RuvA-like domains of EEPD1 markedly decreased co-immunoprecipitation with native Exo1 (Fig. 3*A*). In addition, deletion of the C-terminal regulatory region of Exo1, which does not include the nuclease and DNA binding domains (37), decreased co-immunoprecipitation with native EEPD1 (Fig. 3*B*). Thus, the RuvA domain of EEPD1 may interact with the C-terminal regulatory region of Exo1 (Fig. 3, *A* and *B*) (37). We next tested whether purified recombinant EEPD1 and Exo1 protein could interact *in vitro*. We found that indeed they co-immunoprecipitated when admixed *in vitro* (Fig. 3*C*), indicating that these nucleases interact directly.

##### Exo1 Requires EEPD1 for Efficient End Resection at Stressed Replication Forks

**FIGURE 4.**
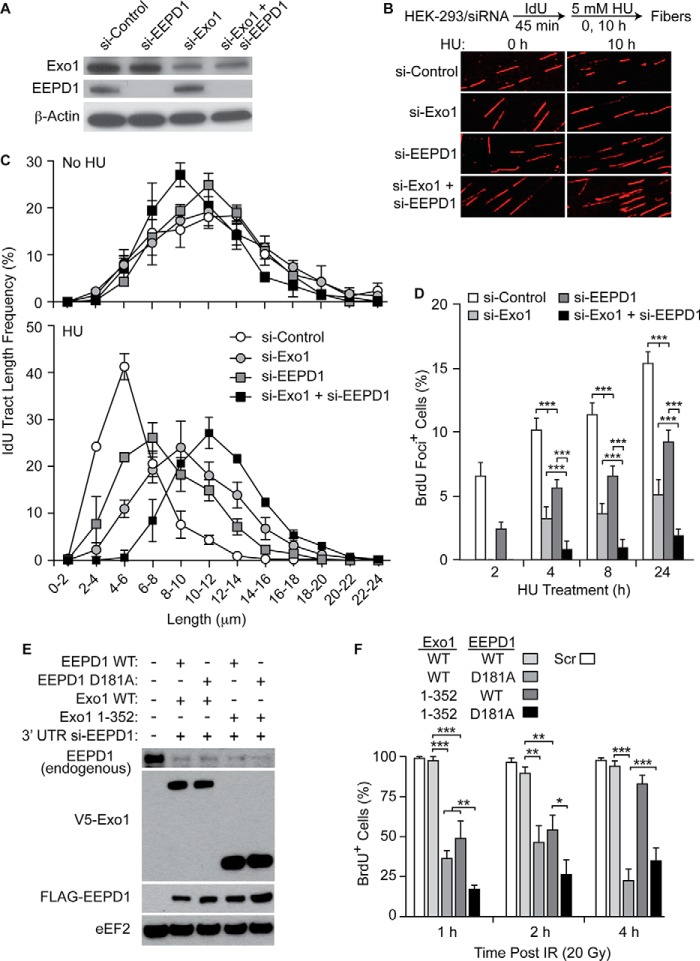
**EEPD1 is required for proper Exo1 5′ end resection at stalled replication fork structures.** *A*, Western analysis of siRNA depletion of Exo1 and EEPD1. *B*, representative images of IdU-labeled nascent DNA fibers before (0 h) and 10 h after hydroxyurea (*HU*) treatment in control HEK-293 cells and cells with depletion of Exo1 and/or EEPD1. *C*, depletion of EEPD1 and/or Exo1 decreases resection of IdU-labeled replication forks during prolonged replication stress (*lower panel*), but has little effect on forks in cells without replication stress (*upper panel*). *D*, time course of end resection *in vivo* measured by assaying BrdU-labeled SS DNA under non-denaturing conditions during replication stress. Depletion of both EEPD1 and Exo1 markedly reduced the presence of end resection at nascent replication forks. Values are means (± S.E.) for 5–6 determinations performed in duplicate. ***, *p* < 0.001, *t* tests. *E*, Western analysis of the depletion of endogenous Exo1 and EEPD1 using siRNA followed by transduction of WT or nuclease-dead mutants of either V5-tagged Exo1 or FLAG-tagged EEPD1. *F*, naked SS BRDU assay of end resection after replication stress with hydroxyurea in the presence of WT or nuclease-dead mutants of Exo1 (D173A) or EEPD1 (D181A). Without EEPD1, there is little end resection after replication stress. *Scr*, scramble; *20 Gy*, 20 grays.

Next, we assessed whether EEPD1 was required along with Exo1 for end resection in cells after prolonged hydroxyurea exposure *in vivo* by measuring degradation of labeled DNA strands at stalled replication forks. Hydroxyurea generates replication stress by stalling forks via nucleotide depletion, and does not directly damage DNA structures. By measuring lengths of nascent DNA replication strands in untreated and hydroxyurea-treated cells (27), we found that depletion of Exo1 and/or EEPD1 had no appreciable effect in untreated cells (Fig. 4, *A*, *B*, and *C*, *upper graph*), but in hydroxyurea-treated cells, nascent replication fibers were longer in cells depleted of Exo1 or EEPD1, indicating reduced resection at stressed replication forks (Fig. 4, *B* and *C*, *lower graph*).

Interestingly, co-depletion of both Exo1 and EEPD1 further increased nascent replication fiber lengths (Fig. 4*C*, *lower graph*), demonstrating that both proteins are needed for appropriate end resection after replication stress. As an alternative approach to measure fork end resection, SS DNA at BrdU-labeled replication forks was measured at intervals after replication stress (31). We found that as replication stress persisted, co-depletion of EEPD1 and Exo1 resulted in significantly less SS DNA at replication forks than when either protein was individually depleted (Fig. 4*D*), confirming that both EEPD1 and Exo1 are needed for optimal end resection at stressed replication forks.

We next tested whether a nuclease-dead EEPD1 mutant could alter the 5′ end resection that initiates HR. Depleting native EEPD1 and transfecting the EEPD1 D181A species that has deficient nuclease activity markedly represses 5′ end resection *in vivo* (Fig. 4, *E* and *F*). This EEPD1 species would likely still interact with Exo1, implying that EEPD1's role is more than just recruitment of Exo1, but that proper 5′ end resection requires EEPD1's nuclease activity as well. The *in vitro* nuclease data above are also consistent with this, where Exo1 lost function in the presence of the nuclease-deficient EEPD1 when compared with native EEPD1 (Fig. 2, *B* and *C*). We next tested whether an Exo1 species that had intact DNA binding and nuclease domains but could not interact with EEPD1 could still mediate 5′ end resection *in vivo*. As shown in Fig. 4, *E* and *F*, we found that end resection was markedly delayed but fully recovered over time. This implies that interaction between Exo1 and EEPD1 improves the rate of end resection.

**FIGURE 5.**
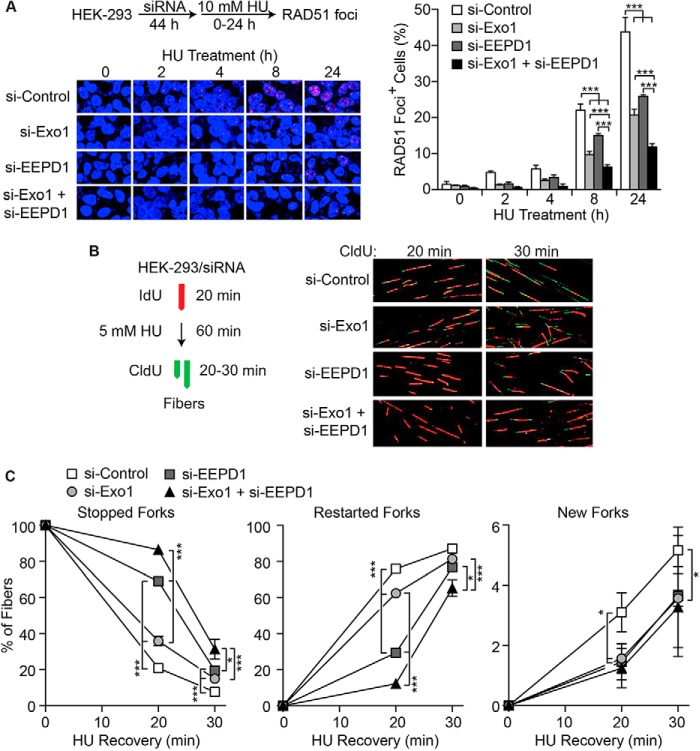
**EEPD1 and Exo1 are both important for replication fork repair.** *A*, *left*, representative confocal immunofluorescence microscopic images of RAD51 foci after hydroxyurea (*HU*) replication stress with or without depletion of EEPD1 and/or Exo1. *Right*, quantitation of RAD51 foci. Values are means (± S.D.) for 3–4 determinations performed in duplicate. ***, *p* < 0.001, *t* tests. *B*, experimental protocol and representative images of DNA fibers from control and EEPD1- and/or Exo1-depleted cells pulse-labeled for 20 min with IdU (*red*), treated with hydroxyurea for 60 min, and then pulse-labeled with CldU (*green*) for 20–30 min. *C*, analysis of replication fork restart failure (*Stopped Forks*) or success (*Restarted Forks*) and initiation of new replication forks by DNA fiber analysis with or with depletion of EEPD1 and/or Exo1. Values are means (± S.D.) for 3 determinations performed in duplicate; >150 fibers were scored per determination. *, *p* < 0.05; **, *p* < 0.01; ***, *p* < 0.001, *t* tests.

Following successful 5′ end resection, 3′ SS DNA is coated with RAD51 to mediate strand invasion (1–3, 10). Thus, the presence of RAD51 foci after replication stress demonstrates successful 5′ end resection. We therefore measured RAD51 foci after replication stress (31) when EEPD1 and/or Exo1 were depleted (Fig. 5*A*). We found that although EEPD1 and Exo1 are each important for RAD51 foci formation after replication stress, depletion of both EEPD1 and Exo1 further decreased RAD51 foci formation (Fig. 5*A*).

DNA fiber analysis in which one fluorochrome is incorporated before replication stress and a second is incorporated when cells are released from stress can be used to assess the restart of stalled replication forks (Fig. 5, *B* and *C*) (27, 31, 33). We used DNA fiber analysis to determine the roles of EEPD1 and Exo1 during stressed replication fork repair and restart. We confirmed our previous observation that depleting EEPD1 increased the percentage of stalled replication forks and reduced fork restart after release from hydroxyurea (31), and a similar result was observed with Exo1 depletion (Fig. 5, *B* and *C*). Depletion of both EEPD1 and Exo1 further exacerbated replication fork recovery after stress when compared with single depletion (Fig. 5*C*). Depletion of EEPD1 decreased fork restart more than depletion of Exo1, consistent with depletion of EEPD1 being more deleterious to end resection than depletion of Exo1 (Figs. 4*F* and 5*C*). EEPD1 and Exo1 depletion individually or together caused slight decreases in new fork initiation after release from hydroxyurea (Fig. 5*C*). These data demonstrate that EEPD1 and Exo1 nucleases have important collaborative functions but distinct functions in replication fork repair and restart.

## Discussion

Previous findings suggest a two-step process for 5′ end resection in HR repair, one short range and another long range (1–3, 36, 38). Mre11 and CtIP are required for initial, short range resection, and are shared between HR and alternative NHEJ (14–16, 26, 39). Mre11 has 3′ overhang endonuclease activity at free DNA ends, and it may assist the long range 5′-exonucleases by clipping off 3′ overhangs to permit access to 5′-recessed ends (28). Mre11 probably has no other role in 5′ end resection, as its major activity is a 3′- to 5′-exonuclease, the opposite of what is required for 5′ end resection (16, 28). Although CtIP may signal initiation of 5′ end resection, its role as an HR nuclease is controversial (39). If short range 5′ end resection does not progress to long range end resection and fork restart via HR, the replication fork may still be rescued by alternative NHEJ (23). However, alternative NHEJ is non-conservative, at best resulting in deletions at the repair junction, and at worst resulting in chromosomal translocations (25). Thus, fork rescue proceeds via HR whenever possible to maintain genomic and functional integrity (1–5). However, cell survival is paramount, and there may be circumstances that cause cells to employ non-HR mechanisms to effect repair of stressed forks. It is also noteworthy that replication fork stress, followed by subsequent collapse, is one of the most poorly tolerated situations cells ever encounter (1–3, 31); therefore, HR repair of stressed replication forks is of fundamental importance.

There are two major exonuclease complexes for long range resection, one based on Exo1 and one with Dna2 (10, 23, 24, 27, 28, 36). Exo1 and Dna2 exist in distinct complexes, both with BLM and RPA, which are likely important for both their nuclease activities (36). Although Dna2 mediates end resection of regressed replication forks (27), many forks may not regress, and these may require cleavage to generate a free 5′ end for resection (1, 2, 31, 38). We previously reported that EEPD1 and Exo1 constitutively co-immunoprecipitate (31). EEPD1 comprises N-terminal RuvA-like and C-terminal DNase I-like domains (31). Here we found that the EEPD1 RuvA domain-containing region was important for Exo1 interaction, whereas EEPD1 interacts with a C-terminal phosphorylated regulatory region of Exo1 (Fig. 3) (37). Exo1 and EEPD1 co-immunoprecipitate *in vitro*, indicating that they directly interact. The constitutive interaction of EEPD1 and Exo1 implies that EEPD1 is a member of the BLM-RPA-Exo1 complex (31, 36).

An Exo1 species with intact nuclease function but that interacted poorly with WT EEPD1 was much less efficient at end resection (Fig. 4*E*). Thus, EEPD1 could promote Exo1 end resection in two ways, first by generating a cleaved replication fork structure that Exo1 can act upon, and second by enhancing Exo1 exonuclease activity. EEPD1's enhancement of Exo1 activity could be from recruitment of Exo1 to the DNA fork structure and/or direct promotion of its nuclease activity (Fig. 2*A*).

The abundance of nucleases that function in HR and replication fork repair raises the question of why so many nucleases are needed. Certainly, there is significant risk of aberrant replication fork structures, and fork repair accuracy is crucial to maintaining genome stability (1–4). It is also likely that HR nucleases function in specific niches, each evolutionarily selected to function in specific circumstances and/or to process specific structures. Given the importance of accurate fork restart for cell survival and genome stability, a degree of redundancy is expected as this would serve to mitigate the effects of reduced function of any individual component. The first step in end resection at stressed but non-regressed replication forks is endonuclease cleavage of the replication fork to generate a double-strand break comprising free 5′ DS ends (1, 6, 7, 9–12). Replication forks can form many structures, but one of the most common and difficult to process is the DS-flap fork structure, where both leading and lagging strands include parental and daughter strands (11, 12). This structure is not cleaved efficiently by Exo1 or Dna2 because it lacks free 5′ ends, and these enzymes act most efficiently by encircling DNA at free ends (28, 40–43).

The parental lagging strand of a duplex Y replication fork structure has a 5′ SS gap at the bifurcation because lagging DNA synthesis must be primed after unwinding. This SS gap in the lagging parental strand is recognized specifically by EEPD1 (Fig. 2, *A* and *B*). This distinguishes EEPD1 from Mus81, which cleaves the leading parental strand at the fork bifurcation (9, 11, 28, 38). Gen1 also has replication fork structure-specific endonuclease activity, but Gen1 is restricted to the cytoplasm until mitosis and thus cannot cleave stressed replication forks during S-phase (30). Interestingly, there is indeed a small amount of cleavage of the leading strand by Exo1 that is manyfold less than the EEPD1-potentiated activity at the lagging strand. This may be due to Exo1's previously described weak flap endonuclease activity (40, 41). It recognizes the leading parental strand as a flap.

We propose that specific nucleases cleave stressed replication forks on the lagging (EEPD1) and leading (Mus81) parental strands (9, 11, 28, 29) to create the required DS end for end resection. Although in mice Mus81 was reported to be essential for replication fork restart (9), Mus81 appears to be dispensable in humans (10). EEPD1, however, is indispensable for proper replication fork restart in human cells (31). It is possible that mouse EEPD1 has reduced function when compared with human EEPD1, or that cleavage of parental lagging strands is more important in humans. In humans, Mus81 could also play an important role in the resolution of Holliday junction structures at the terminal stages of HR repair (9). Thus, there does not appear to be a single fork cleavage enzyme or mechanism across all species by which free DS ends are created at a stalled replication fork to initiate 5′ end resection and HR (11, 12). We propose that EEPD1 fulfills this function in primates.

The additive effect of depleting both EEPD1 and Exo1 on replication fork restart is likely due to the fact that neither is completely depleted by the siRNA. Although the siRNA depletion of each protein was extensive, it was not perfect, and depleting both at the same time may result in a more complete abrogation of the same pathway. Indeed, we do think that although Exo1 and EEPD1 have other distinct roles in DNA repair, in HR repair, we propose that they function in the same pathway, and that the joint incomplete depletion of both produces the additive effects as seen here. Alternatively, it is still possible that EEPD1 has an additional function later in HR, perhaps in resolution of strand invasion.

**FIGURE 6.**
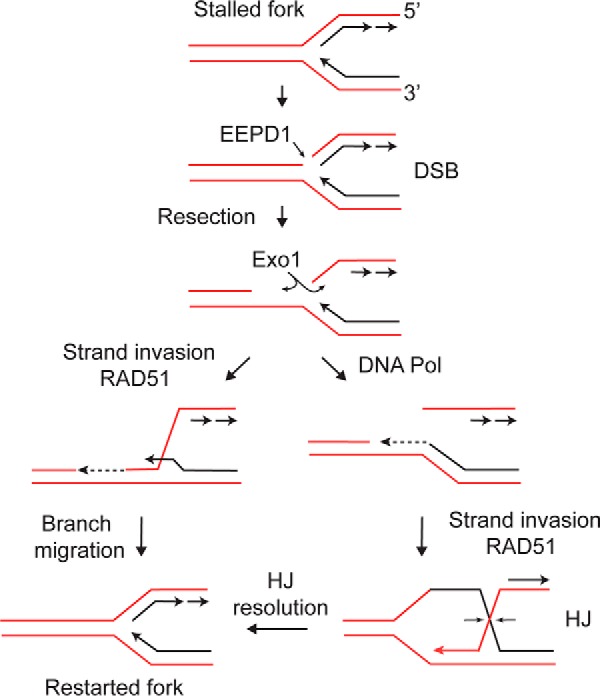
**Model for EEPD1 and Exo1 function in replication stress.** A fork stalled due to nucleotide depletion with hydroxyurea is cleaved by EEDP1, producing a DS end. Either 5′-3′ bidirectional end resection by Exo1 allows direct strand invasion mediated by RAD51 (*left*) to directly restore the fork, or strand invasion occurs subsequent to single-strand gap filling by DNA polymerase (*DNA Pol*), creating a Holliday junction (*HJ*) that is resolved by dual strand cleavage and religation (*horizontal arrows*) to restore the fork. Either HR pathway is error-free and thus maintains genome stability. *DSB*, double-strand break.

Converting a stalled replication fork structure to a free DS end is an essential early step to allow 5′ end resection and fork repair and restart via HR. EEPD1 fulfills several requirements in this process. First, EEPD1 cleaves replication structures (Fig. 2, *B–D*) to create the required free DS end for end resection nucleases (38–43). Second, EEPD1 promotes the activity of Exo1, an important, long range resection nuclease. Third, when Mus81 generates a free DS end at the leading strand, Okazaki fragments must be trimmed and ligated prior to strand invasion; otherwise the template for leading strand synthesis after invasion would be discontinuous. In the EEPD1 pathway, strand invasion by the lagging strand template involves pairing with the leading strand template, both of which are continuous, to reinitiate replication (see model in Fig. 6). Finally, EEPD1 constitutively interacts with Exo1 (Fig. 3), and enhances Exo1 activity (Fig. 2).

This suggests a model in which EEPD1 mediates fork cleavage specifically to initiate HR restart by promoting Exo1-dependent bidirectional resection of the parental and daughter lagging strands (Fig. 2, diagrammed in Fig. 6). It is noteworthy that cleavage of the lagging strands at stressed replication forks can initiate at least two pathways for HR-mediated fork restart (Fig. 6), which would enhance cell survival and genome stability and suppress tumorigenesis (2, 39). One prediction of this model is that EEPD1 depletion should prevent the formation of DS nicks in response to replication stress (38–43). Indeed, when EEPD1 is depleted, we reported that there are fewer DNA nicks induced by hydroxyurea, as measured by comet assay (31).

In summary, we characterized the endonuclease activities of the novel HR component EEPD1 (31), and we show that EEPD1 nuclease activity plays a critical role in promoting Exo1 5′ resection activity to initiate HR-mediated repair of stalled replication forks. We find that both the 5′-endonuclease activity of EEPD1 and the 5′-exonuclease activity of Exo1 are needed for proper end resection at stalled replication forks in human cells. In addition, both nucleases are needed for rapid, efficient restart of stalled replication forks. Thus, EEPD1 is a strong candidate endonuclease for cleavage of the parental lagging strand at stalled, non-regressed replication forks to permit 5′-exonuclease end resection and accurate HR-mediated fork repair and restart.

## Experimental Procedures

### 

#### 

##### Purification of EEPD1 and Exo1

WT and mutant versions of EEPD1 were purified from HEK-293 cells stably expressing FLAG-tagged EEPD1 as we described (32, 33). FLAG-EEPD1 was detected in cell extracts by Western blotting using a FLAG monoclonal antibody (Sigma) as we described previously (32, 33). Cells overexpressing WT or mutant EEPD1 were suspended in 20 ml of Buffer E (50 mm Tris-HCl, pH 7.5, 5 mm DTT, 1% Nonidet-P40, 10% glycerol, 1 mm EDTA), plus mammalian protease inhibitor cocktails containing 0.2 m NaCl, and then centrifuged at 100,000 × *g* for 30 min. Supernatants were filtered through Whatman paper and incubated at 4 °C for 60 min with anti-FLAG affinity gel pre-equilibrated with Buffer E. The beads were washed three times with Buffer E containing 2 m NaCl prior to elution of the protein with Buffer E containing FLAG peptide (500 μg/ml). The eluant was diluted with 10 volumes of Buffer E, and then loaded onto a heparin-Sepharose 6 Fast Flow column (Amersham Biosciences) pre-equilibrated with Buffer E. After washing the column, EEPD1 was fractionated using a linear gradient (0–2 m NaCl) in Buffer E. The eluted protein was dialyzed against Buffer E containing 50 mm NaCl and stored at −80 °C. For preparation of V5-tagged Exo1, cells were harvested and lysed with cold lysis buffer (25 mm HEPES, pH 7.5, 150 mm NaCl, 1.5 mm MgCl_2_, 0.2 mm EDTA, 0.5% Nonidet-P40, 5 μg/ml leupeptin/antipain 1 mm sodium orthovanadate, 1 mm NaF, 1 mm DTT, and 1 mm PMSF) for 30 min at 4 °C, and then clarified by centrifugation. Cell lysates were pre-cleared with protein G-agarose beads (Millipore) for 1 h at 4 °C with rotation prior to the addition of anti-V5 antibody (Invitrogen) for incubation overnight at 4 °C. Protein G-agarose beads (Millipore) were added and incubated for 2 h at 4 °C. After washing eight times with washing buffer (50 mm Tris-HCl, pH 7.5, 1.5 m NaCl, 10% glycerol, 1% Nonidet-P40, and 1 mm EDTA), proteins were eluted with 0.2 m glycine, pH 2.5, into 1.5 m Tris-HCl, pH 8.8. The eluent was dialyzed overnight with dialysis buffer (25 mm Tris-HCl, pH 7.5, 50 mm NaCl, 20% glycerol, 1 mm EDTA, 0.05% Nonidet-P40, 1 mm DTT) and concentrated using Amicon Ultra centrifugal filter units (Millipore).

##### Preparation of ^32^P-Labeled DNA Substrates

DNA substrates were 5′ end-labeled with [γ-^32^P]ATP and T4 polynucleotide kinase as we described (32). DNA substrates were 3′ ^32^P-labeled by incubating 40 pmol of the appropriate SS DNA with 30 units of terminal transferase (Perkin Elmer) in the presence of [α-^32^P]dCTP according to the manufacturer's protocol. The ^32^P-labeled SS DNA was annealed to non-labeled DNAs to prepare indicated DNA substrates for DNA cleavage assay.

##### In Vitro Structure-specific Nuclease Assays

The following oligonucleotides were mixed and annealed to create the DS Y replication fork structure: 5′-CTAGACTCGAGATGTCAAGCAGTCCTAACTTTGAGGCAGAGTCCGTGACGCTCAGTATCG-3′, 5′-CGATACTGAGCGTCACGGACTCTGCCTCAAGACGGTAGTCAACGTGTTACAGACTTGATG-3′, 5′-CATCAAGTCTGTAACACGTTGACTACCGTC-3′, and 5′-GGACTGCTTGACATCTCGAGTCTAG-3′

This results in a daughter lagging strand of 25 nt and a daughter leading strand of 30 nt. The putative unreplicated region is 30 nt. Oligonucleotides used in other structures tested in nuclease assays were described previously (32). DNA cleavage assays were performed using the previously described procedure with modification (33). Briefly, reaction mixtures (20 μl) containing 50 mm Tris-HCl (pH 7.5), 5 mm DTT, 5% glycerol, BSA (2 μg), 2 mm MgCl_2_,0.05% Triton X-100, and 25 mm KCl were incubated with 0.1–0.4 μg of EEPD1 and/or 1.5 ng of Exo1 in the presence of 240 fmol of radiolabeled DNA. In Fig. 1*B*, 50 and 100 ng of native or mutant EEPD1 were used. In Fig. 1*C*, 150 ng of Metnase and 150 or 300 ng of EEPD1 were used. In Fig. 2, *A* and *B*, 20 ng of EEPD1 and 5 ng of Exo1 were used. In Fig. 2, *C–E*, 20 and 40 ng of EEPD1 and 1 ng of Exo1 were used (both native and mutants). Reactions were incubated at 37 °C for the indicated times and products were separated on 12% polyacrylamide gels containing 8 m urea, and detected using a PhosphorImager (GE Healthcare).

##### DNA Resection at Stalled Nascent Replication Forks Measured by Fiber Analysis

Single-label DNA fiber end resection analysis was carried out as described with minor modifications (27). Briefly, HEK-293 cells transfected with the indicated siRNA were grown in 6-well dishes (2 × 10^5^ cells/well), and then 20 μm IdU was added to growth medium and incubated for 45 min at 37 °C. After washing with fresh medium, cells were treated with 5 mm hydroxyurea for 0 or 10 h at 37 °C. Cells were harvested and suspended in PBS, and 1000 cells were transferred to a positively charged microscope slide (Superfrost Plus, Daigger) and processed for DNA fiber analysis as we described previously (33). Slides were mounted in PermaFluor aqueous, self-sealing mounting medium (Thermo Scientific), and DNA fibers were visualized using a confocal microscope (Olympus, FV1000D, 63× oil immersion objective). Images were analyzed using ImageJ software.

##### Immunoprecipitation and Western Analysis

Immunoprecipitation was performed with the Pierce Crosslink Magnetic IP/Co-IP kit according to the manufacturer's instructions (Thermo Scientific catalog number 88805) as we described (31). HEK-293 cells (1 × 10^5^) were collected, washed with PBS, and lysed in a buffer containing 25 mm HEPES (pH 7.5), 0.3 m NaCl, 1.5 mm MgCl_2_, 0.2 mm EDTA, 0.5% Triton X-100, 20 mm β-glycerophosphate, 1 mm sodium vanadate, 1 mm DTT, and protease inhibitor cocktails (Sigma). Cell lysates were loaded onto a SDS-PAGE gel and electrophoresed. Proteins were transferred to a PVDF membrane (Millipore, Billerica, MA) and immunoblotted with primary antibody followed by peroxidase-coupled secondary antibody (Amersham Biosciences) and an enhanced chemiluminescence (Amersham Biosciences) reaction prior to visualization on Kodak-X-Omat film. For the co-immunoprecipitation of FLAG-tagged EEPD1 or V5-tagged Exo1, cells were harvested and washed by PBS before lysis using IP lysis/wash buffer, and then 5 μg of V5 mouse antibody (Invitrogen) were coupled to protein A/G magnetic beads and cross-linked with 20 μm disuccinimidyl suberate. The antibody cross-linked beads were incubated with cell lysate (0.8–1.2 mg) in 500 μl of diluted lysate solution for 1 h at room temperature on a rotator. Beads were collected, washed, and incubated with 100 μl of elution buffer for 5 min at room temperature. Antigen recovery was achieved by collecting the supernatant on a magnetic stand. Protease and phosphatase inhibitors were present in all buffers. *In vitro* co-immunoprecipitation was performed as above except that recombinant tagged EEPD1 and Exo1 were incubated together for 30 min in the above buffer without Triton X-100.

##### DNA End Resection at Nascent Forks Measured by Non-denatured SS BrdU Immunofluorescence

SS naked BrdU DNA replication fork end resection analysis was carried out essentially as we described (31). HEK-293 cells were transfected with si-control, si-Exo1, si-EEPD1, or si-Exo1 with si-EEPD1, and then seeded onto poly-d-lysine coverslips (neuVitro, 18-mm diameter) with fresh medium containing 40 μm BrdU for 36 h. After treatment with 10 mm hydroxyurea for 2–24 h, cells were pre-extracted with 0.5% Triton X-100 and PBS for 5 min on ice and fixed with 4% paraformaldehyde for 20 min. Coverslips were blocked for 1 h with 1% BSA/PBS and incubated with mouse anti-BrdU antibody (1:250; BD Biosciences) overnight in a wet chamber at 4 °C. Coverslips were washed four times, incubated with secondary antibody donkey anti-mouse Alexa Fluor 488 (1:500; Invitrogen) for 1 h, washed four times, and mounted in VECTASHIELD HardSet Mounting Medium with DAPI (Vector Laboratories) on a microscope slide (Fisherfinest Premium Microscope Slides Superfrost®). The samples were analyzed for BrdU foci-positive cells using an Olympus2 confocal microscope with 63× water immersion objective.

##### Rad51 Foci Formation Assay

Immunofluorescent foci formation was assayed as we described (31). HEK-293 cells were seeded onto poly-d-lysine coverslips (neuVitro, 18-mm diameter, catalog number GG-18-PDL) and transfected with control, Exo1, EEPD1, or Exo1/EEPD1 siRNA, and 44–48 h later, cells were treated with 10 mm hydroxyurea for 0–24 h. Cells were pre-extracted with 0.5% Triton X-100/PBS for 5 min on ice and fixed with 4% paraformaldehyde for 20 min. Coverslips were blocked for 1 h with 1% BSA/PBS and incubated with primary rabbit polyclonal anti-Rad51 antibody (1:100; Santa Cruz Biotechnology (H-92) sc-8349) overnight in a wet chamber at 4 °C. Coverslips were washed four times with PBS, incubated with secondary antibody goat anti-rabbit Alexa Fluor 594 (Invitrogen) 1:500 for 1 h, washed four times with TBS, mounted, and analyzed by confocal microscopy as above.

##### DNA Fiber Analysis of Replication Fork Restart

DNA fiber analysis was carried out as we described (31, 33). Briefly, cells were grown in 6-well dishes (2 × 10^5^ cells/well), and then 20 μm IdU was added to growth medium and incubated for 20 min at 37 °C. After washing with fresh medium, cells were treated with 5 mm hydroxyurea for 60 min or mock-treated. Medium was replaced with fresh medium containing 100 μm CldU, and cells were further incubated for the indicated times at 37 °C. Cells were harvested and resuspended in PBS, and 1 × 10^3^ cells were transferred to a positively charged microscope slide (Superfrost/Plus, Daigger), and then processed for DNA fiber analysis as described (33). Slides were mounted in PermaFluor aqueous, self-sealing mounting medium (Thermo Scientific), and DNA fibers were visualized by confocal microscopy.

## Author Contributions

H. S. K. planned and performed experiments, and analyzed data; J. A. N. analyzed data and wrote the manuscript; Y. W. planned and performed experiments E. A. W. planned and performed experiments, and analyzed data; G. S. S. planned and performed experiments; B. L. R. planned and performed experiments; A. S. J. planned and performed experiments; G. S. planned and performed experiments; B. P. planned and performed experiments; K. K. analyzed data; S. B. generated essential reagents and analyzed data; S. H. L. planned experiments, analyzed data, and wrote the manuscript; R. H. planned experiments, analyzed data, and wrote the manuscript.

## Footnotes

3 The abbreviations used are:

HR: homologous recombination
NHEJ: non-homologous end-joining
DS: double-strand
SS: single-strand
nt: nucleotide
IP: immunoprecipitation
CldU: chlorodeoxyuridine
IdU: iododeoxyuridine.

## References

[1] Costes A., and Lambert S. A. (2012) Homologous recombination as a replication fork escort: fork-protection and recovery. Biomolecules 3, 39–71 24970156 10.3390/biom3010039 PMC4030885

[2] Carr A. M., and Lambert S. (2013) Replication stress-induced genome instability: the dark side of replication maintenance by homologous recombination. J. Mol. Biol. 425, 4733–4744 23643490 10.1016/j.jmb.2013.04.023

[3] Zeman M. K., and Cimprich K. A. (2014) Causes and consequences of replication stress. Nat. Cell Biol. 16, 2–9 24366029 10.1038/ncb2897 PMC4354890

[4] Aguilera A., and Gómez-González B. (2008) Genome instability: a mechanistic view of its causes and consequences. Nat. Rev. Genet. 9, 204–217 18227811 10.1038/nrg2268

[5] Allen C., Ashley A. K., Hromas R., and Nickoloff J. A. (2011) More forks on the road to replication stress recovery. J. Mol. Cell. Biol. 3, 4–12 21278446 10.1093/jmcb/mjq049 PMC3030971

[6] Petermann E., and Helleday T. (2010) Pathways of mammalian replication fork restart. Nat. Rev. Mol. Cell Biol. 11, 683–687 20842177 10.1038/nrm2974

[7] Yeeles J. T., Poli J., Marians K. J., and Pasero P. (2013) Rescuing stalled or damaged replication forks. Cold Spring Harb. Perspect. Biol. 5, a012815 23637285 10.1101/cshperspect.a012815 PMC3632063

[8] Arnaudeau C., Lundin C., and Helleday T. (2001) DNA double-strand breaks associated with replication forks are predominantly repaired by homologous recombination involving an exchange mechanism in mammalian cells. J. Mol. Biol. 307, 1235–1245 11292338 10.1006/jmbi.2001.4564

[9] Hanada K., Budzowska M., Davies S. L., van Drunen E., Onizawa H., Beverloo H. B., Maas A., Essers J., Hickson I. D., and Kanaar R. (2007) The structure-specific endonuclease Mus81 contributes to replication restart by generating double-strand DNA breaks. Nat. Struct. Mol. Biol. 14, 1096–1104 17934473 10.1038/nsmb1313

[10] Petermann E., Orta M. L., Issaeva N., Schultz N., and Helleday T. (2010) Hydroxyurea-stalled replication forks become progressively inactivated and require two different RAD51-mediated pathways for restart and repair. Mol. Cell 37, 492–502 20188668 10.1016/j.molcel.2010.01.021 PMC2958316

[11] Rass U. (2013) Resolving branched DNA intermediates with structure-specific nucleases during replication in eukaryotes. Chromosoma 122, 499–515 24008669 10.1007/s00412-013-0431-z PMC3827899

[12] Schwartz E. K., and Heyer W. D. (2011) Processing of joint molecule intermediates by structure-selective endonucleases during homologous recombination in eukaryotes. Chromosoma 120, 109–127 21369956 10.1007/s00412-010-0304-7 PMC3057012

[13] Tay Y. D., and Wu L. (2010) Overlapping roles for Yen1 and Mus81 in cellular Holliday junction processing. J. Biol. Chem. 285, 11427–11432 20178992 10.1074/jbc.M110.108399 PMC2857021

[14] Chapman J. R., Taylor M. R., and Boulton S. J. (2012) Playing the end game: DNA double-strand break repair pathway choice. Mol. Cell 47, 497–510 22920291 10.1016/j.molcel.2012.07.029

[15] Kakarougkas A., and Jeggo P. A. (2014) DNA DSB repair pathway choice: an orchestrated handover mechanism. Br. J. Radiol. 87, 20130685 24363387 10.1259/bjr.20130685 PMC4064598

[16] Symington L. S., and Gautier J. (2011) Double-strand break end resection and repair pathway choice. Annu. Rev. Genet. 45, 247–271 21910633 10.1146/annurev-genet-110410-132435

[17] Bouwman P., Aly A., Escandell J. M., Pieterse M., Bartkova J., van der Gulden H., Hiddingh S., Thanasoula M., Kulkarni A., Yang Q., Haffty B. G., Tommiska J., Blomqvist C., Drapkin R., Adams D. J., et al (2010) 53BP1 loss rescues BRCA1 deficiency and is associated with triple-negative and BRCA-mutated breast cancers. Nat. Struct. Mol. Biol. 17, 688–695 20453858 10.1038/nsmb.1831 PMC2912507

[18] Bunting S. F., Callén E., Wong N., Chen H. T., Polato F., Gunn A., Bothmer A., Feldhahn N., Fernandez-Capetillo O., Cao L., Xu X., Deng C. X., Finkel T., Nussenzweig M., Stark J. M., and Nussenzweig A. (2010) 53BP1 inhibits homologous recombination in *Brca1*-deficient cells by blocking resection of DNA breaks. Cell 141, 243–254 20362325 10.1016/j.cell.2010.03.012 PMC2857570

[19] Callen E., Di Virgilio M., Kruhlak M. J., Nieto-Soler M., Wong N., Chen H. T., Faryabi R. B., Polato F., Santos M., Starnes L. M., Wesemann D. R., Lee J. E., Tubbs A., Sleckman B. P., Daniel J. A., et al (2013) 53BP1 mediates productive and mutagenic DNA repair through distinct phosphoprotein interactions. Cell 153, 1266–1280 23727112 10.1016/j.cell.2013.05.023 PMC3713552

[20] Escribano-Díaz C., Orthwein A., Fradet-Turcotte A., Xing M., Young J. T., Tkáč J., Cook M. A., Rosebrock A. P., Munro M., Canny M. D., Xu D., and Durocher D. (2013) A cell cycle-dependent regulatory circuit composed of 53BP1-RIF1 and BRCA1-CtIP controls DNA repair pathway choice. Mol. Cell 49, 872–883 23333306 10.1016/j.molcel.2013.01.001

[21] Feng L., Fong K. W., Wang J., Wang W., and Chen J. (2013) RIF1 counteracts BRCA1-mediated end resection during DNA repair. J. Biol. Chem. 288, 11135–11143 23486525 10.1074/jbc.M113.457440 PMC3630874

[22] Zimmermann M., Lottersberger F., Buonomo S. B., Sfeir A., and de Lange T. (2013) 53BP1 regulates DSB repair using Rif1 to control 5′ end resection. Science 339, 700–704 23306437 10.1126/science.1231573 PMC3664841

[23] Truong L. N., Li Y., Shi L. Z., Hwang P. Y., He J., Wang H., Razavian N., Berns M. W., and Wu X. (2013) Microhomology-mediated end joining and homologous recombination share the initial end resection step to repair DNA double-strand breaks in mammalian cells. Proc. Natl. Acad. Sci. U.S.A. 110, 7720–7725 23610439 10.1073/pnas.1213431110 PMC3651503

[24] Zhu Z., Chung W. H., Shim E. Y., Lee S. E., and Ira G. (2008) Sgs1 helicase and two nucleases Dna2 and Exo1 resect DNA double-strand break ends. Cell 134, 981–994 18805091 10.1016/j.cell.2008.08.037 PMC2662516

[25] Wray J., Williamson E. A., Singh S. B., Wu Y., Cogle C. R., Weinstock D. M., Zhang Y., Lee S. H., Zhou D., Shao L., Hauer-Jensen M., Pathak R., Klimek V., Nickoloff J. A., and Hromas R. (2013) PARP1 is required for chromosomal translocations. Blood 121, 4359–4365 23568489 10.1182/blood-2012-10-460527 PMC3663429

[26] Zhang Y., and Jasin M. (2011) An essential role for CtIP in chromosomal translocation formation through an alternative end-joining pathway. Nat. Struct. Mol. Biol. 18, 80–84 21131978 10.1038/nsmb.1940 PMC3261752

[27] Thangavel S., Berti M., Levikova M., Pinto C., Gomathinayagam S., Vujanovic M., Zellweger R., Moore H., Lee E. H., Hendrickson E. A., Cejka P., Stewart S., Lopes M., and Vindigni A. (2015) DNA2 drives processing and restart of reversed replication forks in human cells. J. Cell Biol. 208, 545–562 25733713 10.1083/jcb.201406100 PMC4347643

[28] Tsutakawa S. E., Lafrance-Vanasse J., and Tainer J. A. (2014) The cutting edges in DNA repair, licensing, and fidelity: DNA and RNA repair nucleases sculpt DNA to measure twice, cut once. DNA Repair 19, 95–107 24754999 10.1016/j.dnarep.2014.03.022 PMC4051888

[29] Sarbajna S., Davies D., and West S. C. (2014) Roles of SLX1-SLX4, MUS81-EME1, and GEN1 in avoiding genome instability and mitotic catastrophe. Genes Dev. 28, 1124–1136 24831703 10.1101/gad.238303.114 PMC4035540

[30] Chan Y. W., and West S. C. (2014) Spatial control of the GEN1 Holliday junction resolvase ensures genome stability. Nat. Commun. 5, 4844 25209024 10.1038/ncomms5844 PMC4172962

[31] Wu Y., Lee S. H., Williamson E. A., Reinert B. L., Cho J. H., Xia F., Jaiswal A. S., Srinivasan G., Patel B., Brantley A., Zhou D., Shao L., Pathak R., Hauer-Jensen M., Singh S., et al (2015) EEPD1 rescues stressed replication forks and maintains genome stability by promoting end resection and homologous recombination repair. PLoS Genet. 11, e1005675 26684013 10.1371/journal.pgen.1005675 PMC4684289

[32] Beck B. D., Lee S. S., Williamson E., Hromas R. A., and Lee S. H. (2011) Biochemical characterization of Metnase's endonuclease activity and its role in NHEJ repair. Biochemistry 50, 4360–4370 21491884 10.1021/bi200333k PMC3388547

[33] Kim H.-S., Chen Q., Kim S.-K., Nickoloff J. A., Hromas R., Georgiadis M. M., and Lee S.-K. (2014) The DDN catalytic motif is required for Metnase functions in NHEJ repair and replication restart. J. Biol. Chem. 289, 10930–10938 24573677 10.1074/jbc.M113.533216 PMC4036204

[34] De Haro L. P., Wray J., Williamson E. A., Durant S. T., Corwin L., Gentry A. C., Osheroff N., Lee S. H., Hromas R., and Nickoloff J. A. (2010) Metnase promotes restart and repair of stalled and collapsed replication forks. Nucleic Acids Res. 38, 5681–5691 20457750 10.1093/nar/gkq339 PMC2943610

[35] Syeda A. H., Hawkins M., and McGlynn P. (2014) Recombination and replication. Cold Spring Harb. Perspect. Biol. 6, a016550 25341919 10.1101/cshperspect.a016550 PMC4413237

[36] Nimonkar A. V., Genschel J., Kinoshita E., Polaczek P., Campbell J. L., Wyman C., Modrich P., and Kowalczykowski S. C. (2011) BLM-DNA2-RPA-MRN and EXO1-BLM-RPA-MRN constitute two DNA end resection machineries for human DNA break repair. Genes Dev. 25, 350–362 21325134 10.1101/gad.2003811 PMC3042158

[37] Tomimatsu N., Mukherjee B., Hardebeck M. C., Ilcheva M., Camacho C. V., Harris J. L., Porteus M., Llorente B., Khanna K. K., and Burma S. (2014) Phosphorylation of EXO1 by CDKs 1 and 2 regulates DNA end resection and repair pathway choice. Nat. Commun. 5, 3561 24705021 10.1038/ncomms4561 PMC4041212

[38] Berti M., and Vindigni A. (2016) Replication stress: getting back on track. Nat. Struct. Mol. Biol. 23, 103–109 26840898 10.1038/nsmb.3163 PMC5125612

[39] Makharashvili N., Tubbs A. T., Yang S. H., Wang H., Barton O., Zhou Y., Deshpande R. A., Lee J. H., Lobrich M., Sleckman B. P., Wu X., and Paull T. T. (2014) Catalytic and noncatalytic roles of the CtIP endonuclease in double-strand break end resection. Mol. Cell 54, 1022–1033 24837676 10.1016/j.molcel.2014.04.011 PMC4079050

[40] Lee B.-i., Nguyen L. H., Barsky D., Fernandes M., and Wilson D. M. 3rd (2002) Molecular interactions of human Exo1 with DNA. Nucleic Acids Res. 30, 942–949 11842105 10.1093/nar/30.4.942 PMC100345

[41] Keijzers G., Bohr V. A., and Rasmussen L. J. (2015) Human exonuclease 1 (Exo1) activity characterization and its function on flap structures. Biosci Rep. 35, e00206 26182368 10.1042/BSR20150058 PMC4613700

[42] Stewart J. A., Campbell J. L., and Bambara R. A. (2010) Dna2 is a structure-specific nuclease, with affinity for 5′-flap intermediates. Nucleic Acids Res. 38, 920–930 19934252 10.1093/nar/gkp1055 PMC2817469

[43] Orans J., McSweeney E. A., Iyer R. R., Hast M. A., Hellinga H. W., Modrich P., and Beese L. S. (2011) Structures of human exonuclease 1 DNA complexes suggest a unified mechanism for nuclease family. Cell 145, 212–223 21496642 10.1016/j.cell.2011.03.005 PMC3093132

